# A study on relapse/re-infection rate of *Plasmodium vivax* malaria and identification of the predominant genotypes of *P. vivax* in two endemic districts of Nepal

**DOI:** 10.1186/1475-2875-12-324

**Published:** 2013-09-16

**Authors:** Sulochana Manandhar, Chop L Bhusal, Umesh Ghimire, Shankar P Singh, Dibesh B Karmacharya, Sameer M Dixit

**Affiliations:** 1 Center for Molecular Dynamics Nepal, 5th Floor Swaraj Sadan, Prasuti Griha Marg, Thapathali-11, Kathmandu, Nepal; 2 Nepal Health Research Council, Ministry of Health Complex, Ramshahpath, Kathmandu, Nepal

**Keywords:** *Plasmodium vivax*, Circumsporozoite protein, Genotypes, Relapse/reinfection rate, Nepal

## Abstract

**Background:**

Malaria is a major public health problem in Nepal inflicted primarily by the parasite *Plasmodium vivax*, - the only species responsible for relapse cases in Nepal. Knowledge on its relapse rate is important for successful malaria control, but is lacking in Nepal. The information on circulating predominant genotypes of *P. vivax* is equally relevant for high endemic districts of Nepal to understand the transmission dynamics of the parasite and to uncover the coverage and efficacy of potential vaccine beforehand.

**Methods:**

A prospective observational study with a six months follow-up period was conducted from August 2010 to May 2011 in four health centres of Kailali and Kanchanpur districts of Nepal to access the relapse/re-infection rate of *P. vivax*. The prevalence and heterogeneity of its genotypes were identified by PCR-RFLP assay targeting central repeat region of circumsporozoite protein (*Pvcsp*).

**Results:**

In total, 137 cases microscopically suspected to have *P. vivax* infection were enrolled in the study. Of these, 23 cases (17%) were detected for the relapse/ re-infection-during a six-month period, with a high proportion being male cases of age group 11–20 years. For genotyping, 100 whole blood samples were analysed, of which 95% of the parasite isolates were found to be of VK210 genotype. The minor genotype VK247 existed either in isolation or as mixed infection with VK210 in rest of the samples.

**Conclusions:**

The relapse/re-infection rate of 17% was determined for *P. vivax* in Kailali and Kanchanpur districts of Nepal. A heterogeneous *Pvcsp* genotypic distribution of *P. vivax* was detected with VK210 being a predominant type, suggesting a complex transmission dynamics of the parasite. Expanding such study in other endemic regions of Nepal would help provide a complete picture on relapse/re-infection rate and parasite genotypic variability that can help in effective control and management of malaria in Nepal.

## Background

In Nepal, 22.5 million people still live in malaria-prone areas with 65 of 75 districts considered endemic to the disease [1]. Malaria control has been identified as priority-I public health programme of Nepal under the National Health Sector Programme Implementation Plan-II (NHSP-II) 2010–2015 [2]. In Nepal, *Plasmodium vivax* is responsible for most of disease burden, with 75 to 80% of reported malaria cases being accounted by this species of the parasite [2]. It is also the only species in Nepal responsible for leading to relapses. It is co-endemic with *Plasmodium falciparum* in high-endemic districts [1]. Since mortality due to *P. vivax* is lower compared to *P. falciparum*, the former species has been relatively neglected and, therefore, largely under-researched [3]. Controlling vector-borne diseases, such as malaria, is a challenge for economically-weak countries like Nepal. Further, the possibility of relapse infections exhibited in the cases of *P. vivax* is a barrier to successful treatment and control of malaria. Thus, determining relapse pattern is an indispensible component for early management of vivax malaria.

The neighbouring cities of Indian states are not only the hub of employment for Nepalis, especially those residing near the border, but are also the source of infectious diseases. A significant proportion of malaria in the eastern region of Nepal and all other bordering districts is considered to be imported from border cities of India [4]. Since malarial epidemiology varies considerably between different geographic regions as a result of complex interplay among humans and vectors, such porous borders impose a significant contribution in affecting the genetic complexity of the parasite. The knowledge on genotypes of malarial parasites contributes greatly to our understanding of the dynamics of disease transmission because studies have shown probable difference in the choice of preferred *Anopheles* species as a vector by various genotypes of *P. vivax*[5]. Genotyping of *P. vivax* species can also help differentiate between a new infection and a relapsed one, which can play an important role in controlling the disease [6]. Since several antigens expressed on the surface of the parasite or on infected human blood or liver cells strongly illicit the host immune system, any genetic polymorphisms in these antigens play a critical role in evading protective immune responses thereby hindering the development of an effective vaccine against any *Plasmodium* species [7]. Thus, understanding the genetic diversity of potent surface antigens of *P. vivax* from various endemic geographic regions across the world is important in providing crucial data for development of an effective vaccine [8].

Circumsporozoite protein (*Pvcsp*) is an abundant surface antigen of *P. vivax* sporozoites. The gene encoding this protein contains a central region composed of one of the two types of nona-peptide repeat units, based on which, the parasite has been divided into two genotypic variants. The variants with one of the repeat units GDRA(A/D)GQPA, that is restriction digested by the enzyme *Alu*I is referred to as VK210 genotype while those with the other repeat unit ANGA(G/D)(N/D)QPG, that is restricted by *BstN*I is recognized as VK247 type [9-12]. Studies have showed differential geographical distribution and probable differences in transmission intensity, vector selection, drug resistance and treatment responses existing in these genotypes [13-16].

Since no *Pvcsp-*based genotyping studies have been carried out before in Nepal, this study aims to provide a pilot data on *Pvcsp* genotypes circulating in Kailali and Kanchanpur districts for Nepal.

## Methods

### Relapse/re-infection study

A prospective observational study was carried out in four health centres of Kailali and Kanchanpur districts of far-west Nepal from August 2010 to May 2011. These districts are among high malaria endemic districts of Nepal. As per the 2010 annual report of Department of Health Services, Government of Nepal, the annual parasite incidence rate per 1,000 population was 1.77% for Kailali and 0.6% for Kanchanpur ,while that for the entire nation was just 0.16% in the year 2010.

After taking an informed consent from malaria-suspected symptomatic patients and performing their initial clinical examination, the blood samples were collected for thick blood film microscopic examination. The parasitological examination was done independently by two trained laboratory technicians and the slides were sent to an independent laboratory for quality assurance. All febrile patients positive for *P. vivax* in microscopy at the time of visit and confirming to be able to come on stipulated follow-up visits were considered eligible and were enrolled in the study. Any of the cases failing to show the microscopic evidence of *P. vivax* or showing mixed or isolated infection with *P. falciparum* or presenting any feature of severe malaria or any other underlying chronic severe illness were excluded from the study. Pregnant women and lactating mothers were also excluded from the study.

The *P. vivax*-confirmed enrolled cases were asked to complete the three-day 25 mg/kg body weight of chloroquine therapy. The national anti-malarial treatment guidelines of Nepal suggested the combined use of three-day chloroquine and 14-day primaquine for all *P. vivax* confirmed cases. However, the actual trend practiced in the selected study area was to provide chloroquine monotherapy to all cases of clinical and *P. vivax* malaria. The chloroquine-primaquine therapy was administered only in the cases who returned to the centre with renewed symptoms of malaria and further microscopic evidence of *P. vivax*.

The first dosage of chloroquine was administered under direct observation of the research team on the day when the participant was first enrolled in the study. The second and third dosages were prescribed for home use with a detail instruction on the drug regimen and follow up and with a proper education on the importance of doing so.

The cases were advised to come for follow-up visits on the third day and at the sixth month of enrolment or on any day/s when the patient felt febrile. The cases were rigorously reminded for follow up visits by regular telephone calls. When required, home visits were also done by the trained and locally mobilized community field team. An absolute treatment compliance was assumed to have achieved if the cases succeeded to appear in all stipulated follow up days.

Because the selected study areas had high *P. vivax* incidence rate, the chances of re-infection at any point after the completion of chloroquine therapy could not be ignored. Thus, all recurrences were assumed to be either because of relapses or re-infections. And because the study areas had rare history of treatment failure for *P. vivax* chloroquine therapy, the recrudescence was assumed to be unlikely.

Due to the logistical and financial constrains, the cases could be followed up for a period of six months only. On each follow-up day, thick blood film examination was carried out. Those febrile patients who returned the centre after one month of the start of chloroquine therapy and were microscopically found to be positive for *P. vivax* were considered relapse/re-infection cases whose rate was determined by dividing the number of such recurred cases by total number of *P. vivax* confirmed cases treated with standard chloroquine regimen. The cases presenting with recurrences and the remainder of enrolled cases who completed six months of follow up were given primaquine therapy in the dosage of 7.5 mg twice a day for 14 days after screening for G6PD deficiency.

### Genotyping study

Parasite DNA was extracted from whole blood samples using commercial DNA extraction kit (QiagenDNeasy Blood & Tissue Kit). Before processing for genotyping assay, all microscopically detected samples were confirmed for the presence of *P. vivax* by two rounds of nested PCR targeting ssrRNA [17]. *Plasmodium vivax* confirmed samples were genotyped by nested PCR-RFLP assay based on central repeat region of *Pvcsp* gene as mentioned by Imwong *et al.*[18]. Briefly PCR reaction was set with 800 nM dNTPs, 1 mM MgCl2, 0.4 unit Taq polymerase, 400 nM of each primers with 1 μl of DNA template for first round and 1 μl of first round PCR product as template for second round nested PCR. The thermocycling condition for first round PCR was 95°C for 5 min followed by 25 cycles of 58°C for 2 min, 72°C for 2 min and 94°C for 1 min, and one cycle of each 58°C for 2 min and 72°C for 5 min. That for second nested PCR was exactly the same as for first round except that annealing was at 62°C for 2 min and with 30 cycles. The second round products were digested by restriction enzymes *Alu*I and *BstN*I in parallel set of tubes and visualized in 2% agarose gel to distinguish between two genotypes of *P. vivax* on the basis of susceptibility to digestion by any one of the enzymes*.* Broader *Pvcsp* genotypes of *P. vivax* isolates were further attempted to subdivide into finer *Pvcsp* allelic types utilizing fragment size heterogeneity of PCR products and sequence variations in pre- and post-repeat regions by PCR-RFLP protocol followed by 3% agarose gel electrophoresis as mentioned by Imwong *et al.*[18].

### Ethical approval

This study was initiated by Nepal Health Research Council (NHRC), with documented approval from its Ethical Review Board (ERB).

## Results

### Relapse/re-infection assessment

**Figure 1:**
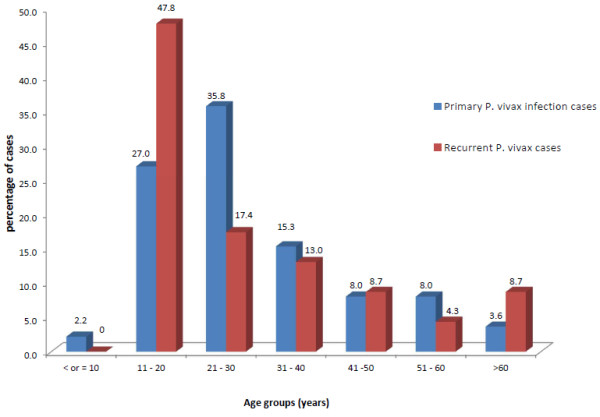
**Percentage distribution of primary and recurrent infection cases of *Plasmodium vivax *among different age groups.**

Among malaria-suspected patients visiting four selected health centres, a total of 137 cases were microscopically observed for *P. vivax* infection and fulfilled other inclusion criteria. Age distribution of these cases varied from 15 to 83 years. The highest percentage of cases affected belonged to the productive age group of 21 to 30 years (35.7%) followed by 11 to 20 years (27%) (Figure 1). The number of male cases affected was much higher (81%) than that of female cases. Out of 137 total cases enrolled, recurrence was reported among 23 cases (17%). A high percentage (47.8%) of relapse/re-infection occurred among young people of age group of 11 to 20 years (Figure 1) and among male patients (91.3%). However this recurrence rate difference by gender could not be considered significant because the initial number of male cases studied was much greater than that of female cases.

All of the 23 cases of the first reported recurrences occurred after one month of primary detection of the parasite. During a six-month follow-up period, most (56.56%) recurrences were reported only once. The remainder of the recurrences were reported for multiple number of times ranging from twice to five times despite the possible completion of 14-day unobserved primaquine therapy.

### Genotype assessment

**Figure 2:**
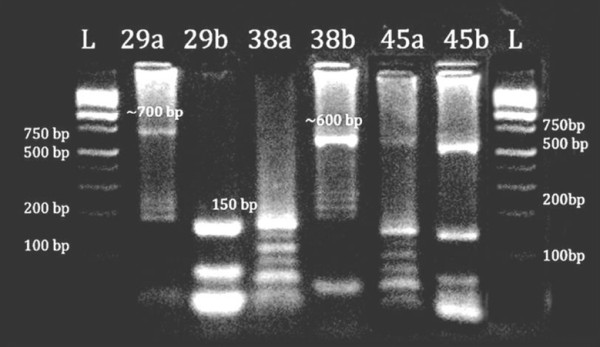
**Gel picture of PCR RFLP-based *Pvcsp *genotyping of *Plasmodium vivax *for samples 29, 38 and 45; a and b are AluI and BstNI digestion products for each samples.** Samples 29 and 38 each were digested by BstNI and AluI only, respectively, suggesting them to be of VK247 and VK210 type, respectively. Sample 45 was digested by both AluI and BstNI, suggesting it to be a mixed infection.

Molecular analysis could be carried out in only 100 randomly selected blood samples. All of these samples were positive for *P. vivax* in two separate rounds of nested PCR. Among all samples tested, 95% of *P. vivax* parasites were found to be of VK210 genotype in PCR-RFLP based *Pvcsp* molecular genotyping assay. The remainder (5%) of the isolates belonged to VK247 type, 40% of which existed as mixed infection with VK210. Figure 2 shows representative agarose gel image of restriction digestion of *Pvcsp* gene for genotyping test.

Allelic variations of each VK210 and VK247 isolates could not be distinguished either based on fragment size variation of PCR products or PCR-RFLP based sequence variation of pre and post repeat regions when analysed in 3% agarose gel.

## Discussion

It has been proposed that in endemic areas, a large proportion of indigenous population harbours latent hypnozoite forms of *P. vivax,* which can be activated by a systemic illness such as *vivax* or *falciparum* malaria. This is the reason for observed higher rates of relapse in people living in endemic areas especially in the pockets infiltrated with both of these malarial species [19]. The relapse rate of *P. vivax* varies considerably across various geographic regions. It also depends on immunity of the subjects to the disease [19,20]. Such relapses pose significant hurdles in an effective control of malaria, particularly for endemic countries like Nepal. However, in context of Nepal, negligible research has been conducted addressing such problems.

Since the two districts chosen for this study had high incidence of *P. vivax* transmission, higher probability of renewed infection among the enrolled cases could be expected at any time after completion of the chloroquine therapy in a developing country settings. Due to the nature of the study, re-infections could not be differentiated from relapses in this study and as such all recurrences were unequivocally considered as either relapse or re-infection. However, because primaquine was excluded initially for the treatment of *P. vivax* confirmed enrolled cases, it is highly probable that majority of recurrences were due to relapses.

In this study, a stringent follow up schedule had been practiced ensuring an absolute adherence to chloroquine treatment. Further, all study subjects were observed to respond well to the therapy thereby excluding any incidences of treatment failure. Moreover because all first time reported recurrences occurred after 28 days of primary detection and completion of standard chloroquine therapy, the possibility of recrudescence could be excluded in this study.

During six months’ follow-up, a relapse/re-infection rate of 17% was detected. Variable recurrence rates of *P. vivax* has been recorded across the globe. In India, prospective studies carried out over the past 25 years have recorded post-chloroquine anti-malarial treatment recurrence rates to vary between 8.6% (Orissa), 8.9% (Madhya Pradesh) [19], 12.6% (Mumbai) [21] and 40% (Delhi) [22]. Variable latency period among *P. vivax* phenotypes has been described [19,23]. A study in Delhi, India has observed a distinct rise in percentage of relapse cases as the follow-up period increased from one year (23.3%) to five years (44.3%) [22]. These findings suggest that determination of an actual relapse rate relies greatly on the duration of follow up. Taking this into consideration, for the follow-up period being less than a year, the relapse/re-infection rate of 17% observed in this study might probably be underestimated. Further, because there might possibly be many asymptomatic self- limiting relapse/re-infection cases that might have gone unreported and thus failed being recaptured, the true relapse/re-infection rate might have been higher than observed here.

A high percentage of relapse/reinfection was recorded among young people of age group 11 to 20 years which appeared to decrease with increasing age. This corroborates with literature findings that some degree of immunity is gained by early adulthood in indigenous population living in endemic areas, thereby reducing the number of relapses in increasing age groups [19].

In this study, a 14-day standard primaquine therapy was prescribed for all confirmed recurrence cases. However, many cases repeatedly suffered further episodes of recurrences, ranging from twice to as many as five times within a half-year period from primary infection. This suggested that the prescribed primaquine regimen as per the WHO recommendations [24] possibly was not absolutely efficient for preventing all relapses. This kind of therapeutic response variability and inadequacy has also been observed in many endemic regions [20,25,26]. However, it must be pointed out that, since the primaquine treatment provided to study subjects was an unmonitored 14-day course, an absolute compliance cannot be assured. Further, many of the recurrences might have been due to the renewed infection by blood stage parasites, against which the primaquine has reduced effect.

Regarding the circumsporozoite genotyping, VK210 genotype was found to be the most predominant genotype constituting 95% of parasite isolates. Though global prevalence of individual genotypes varies geographically, such prepotency of the VK210 genotype of *P. vivax* has been a common worldwide occurrence [27,28]. A similar study carried out in Pakistan showed results almost identical to this study - reporting isolation of 95.7% of VK210, 2.7% of VK247 and 1.6% of mixed genotypes. Such VK210 predominance has been reported in other malaria-endemic countries such as Myanmar (66%), Thailand (77%), Brazil (86%), Kolkata (India) (99.3%) Azerbaijan (100%) and Honduras (100%) [5,8,13,29-31]. This is in contrast to the findings of some studies reporting the predominance of VK247 genotype [32-34]. Such differences in prevalence of a different variant of parasite could be due to the predominance of different species and strain of *Anopheles* mosquito vector that is more susceptible to VK247 genotype as mentioned in several studies [14]. One study in southern Mexico [35] showed that *Anopheles albimanus* was more susceptible to infections by the VK210 subtype, while *Anopheles pseudopunctipennis* was more susceptible to VK247. However, in context of Nepal, out of 42 *Anopheles* species found, only three species, *Anopheles fluviatilis, Anopheles maculates* and *Anopheles annularis* have been identified as vectors of malaria [2]. This suggests that there might exist a unique dynamic interaction among existing *P. vivax* genotypes and Nepali strains of *Anopheles* vectors, thereby warranting further studies to verify this hypothesis.

Allelic variation of each of VK210 and VK247 based on number of repeats of central motif of *Pvcsp* gene and thus differences in size of PCR products could not be detected as previously observed [11,25] either because the 3% agarose gel was not sufficient enough to resolve the existing fragment length variations, or isolates in this study lacked this variation. Further, PCR-RFLP analysis of the isolates based on pre- and post repeats adjoining the central repeat region could not be determined when restriction-digested products were run in 3% agarose gel probably because of the same above reasons. Use of capillary electrophoresis might have provided enough resolution to identify these minor fragment length variations.

## Conclusion

In conclusion, *P. vivax* relapse/re-infection rate of 17% was observed during a brief follow up of six months, suggesting that the actual rate could be even higher provided the follow up were to extend. This contributes a significant hurdle in effective control of malaria in Nepal and challenges its ambitious goal to see a malaria-free country by 2026.

Since this is the first-ever *Pvcsp* marker-based genotyping study carried out in Nepal, predominance of VK210 genotype can be considered as a primary information on *Pvcsp* genotype for Nepal. Further, as this study involved only two out of 65 malaria-endemic districts of Nepal, a larger representative study is warranted to provide further evidence on the type(s) and distribution of the prevailing *Pvcsp* genotypes of *P. vivax*.

## Abbreviations

PCR: Polymerase chain reaction; Pvcsp: *Plasmodium vivax* circumsporozoite protein; RFLP: Restriction fragment length polymorphism.

## Competing interests

The authors declared that they have no competing interests.

## Authors’ contributions

SM: all laboratory procedures including development of manuscript; CLB: Initiated initiated research as the Head of NHRC, the funding body and also supported MS preparation; UG: all fieldwork leading to collection of samples, and help in writing parts of manuscript; DK: supported laboratory work, and manuscript preparation; SPS: advisory role in manuscript preparation and field research; SMD: coordinated research and manuscript preparation. All authors read and approved the final manuscript.
